# Emergence and Dissemination of Extraintestinal Pathogenic High-Risk International Clones of *Escherichia coli*

**DOI:** 10.3390/life12122077

**Published:** 2022-12-10

**Authors:** Béla Kocsis, Dániel Gulyás, Dóra Szabó

**Affiliations:** Institute of Medical Microbiology, Semmelweis University, H-1089 Budapest, Hungary

**Keywords:** multidrug resistance, nosocomial infections, *E. coli* high-risk clones, whole-genome-sequencing

## Abstract

Multiresistant *Escherichia coli* has been disseminated worldwide, and it is one of the major causative agents of nosocomial infections. *E. coli* has a remarkable and complex genomic plasticity for taking up and accumulating genetic elements; thus, multiresistant high-risk clones can evolve. In this review, we summarise all available data about internationally disseminated extraintestinal pathogenic high-risk *E. coli* clones based on whole-genome sequence (WGS) data and confirmed outbreaks. Based on genetic markers, *E. coli* is clustered into eight phylogenetic groups. Nowadays, the *E. coli* ST131 clone from phylogenetic group B2 is the predominant high-risk clone worldwide. Currently, strains of the C1-M27 subclade within clade C of ST131 are circulating and becoming prominent in Canada, China, Germany, Hungary and Japan. The C1-M27 subclade is characterised by *bla*_CTX-M-27._ Recently, the ST1193 clone has been reported as an emerging high-risk clone from phylogenetic group B2. ST38 clone carrying *bla*_OXA-244_ (a *bla*_OXA-48-like_ carbapenemase gene) caused several outbreaks in Germany and Switzerland. Further high-risk international *E. coli* clones include ST10, ST69, ST73, ST405, ST410, ST457. High-risk *E. coli* strains are present in different niches, in the human intestinal tract and in animals, and persist in environment. These strains can be transmitted easily within the community as well as in hospital settings. WGS analysis is a useful tool for tracking the dissemination of resistance determinants, the emergence of high-risk mulitresistant *E. coli* clones and to analyse changes in the *E. coli* population on a genomic level.

## 1. Introduction

*Escherichia coli* is a Gram-negative rod-shaped commensal bacterium in the human intestine; however, it is also a major causative agent of several infections. Extraintestinal pathogenic *E. coli* (ExPEC) is responsible for a wide range of severe community- and hospital-acquired infections, such as neonatal meningitis, peritonitis, and bloodstream and urinary tract infections (UTI) [1,2]. Furthermore, multiresistant *E. coli* strains are responsible for a high number of hospital outbreaks worldwide that are associated with longer hospital stays, increased health care costs and high mortality rates [3,4,5]. 

*E. coli* has a remarkable capacity to take up and to accumulate various genetic materials, including plasmids, integrons, and transposons through horizontal gene transfer; thus, *E. coli* can acquire different antibiotic resistance genes, enabling it to develop multiresistance [6,7]. Currently, extended-spectrum beta-lactamase (ESBL)- and carbapenemase-producing *E. coli* are of great concern worldwide [8,9]. The most frequently detected ESBLs in *E. coli* clinical isolates are mainly CTX-M-type enzymes [10,11]; however, transferable genetic elements harbouring carbapenemase genes have also been described, namely, *bla*_NDM_ (New Delhi metallo-β-lactamase), *bla*_KPC_ (*Klebsiella pneumoniae* carbapenemase) and *bla*_OXA-48_ (OXA-48 carbapenemase) [12,13,14,15,16,17]. Further antibiotic resistance genes have also been reported in *E. coli* clinical isolates, namely, fluoroquinolone-, aminoglycoside- and colistin-resistance determinants [18,19,20].

Carbapenem- and third-generation cephalosporin-resistant Enterobacteriaceae are grouped into the ‘critical’ category on the priority list of the World Health Organisation (WHO); therefore, there is an urgent need for effective antibiotics against this pathogen [21]. Some novel antibiotics have already been approved and marketed in recent years to combat multiresistant *E. coli* infections. Among these recently marketed agents, we find beta-lactam plus beta-lactamase inhibitors (e.g., ceftazidime + avibactam, ceftolozane + tazobactam, meropenem + vaborbactam), fluoroquinolone (e.g., delafloxacin), aminoglycoside (e.g., plazomicin) agents [22,23,24]. Apart from novel antibiotics, some synergistic antibiotic combinations are also available to treat infections caused by multiresistant *E. coli* [25,26].

Multiresistant *E. coli* strains possess a remarkably complex genomic plasticity; therefore, they can adapt to different conditions and persist in diverse hosts; moreover, they can be easily transmitted between different hosts. According to the‘One Health’ approach in antimicrobial resistance, *E. coli* has different reservoirs (human, animal and environmental) and the accumulation of resistance genes can take place in all of the different reservoirs [27,28]. Furthermore, multiresistant *E. coli* strains can be selected out in human, animal, and environmental niches, resulting in the development of high-risk multiresistant *E. coli* clones. High-risk clones sustain their fitness, and their dissemination locally or even globally is associated with high variability of resistance and virulence genes [29,30,31,32].

**Figure 1:**
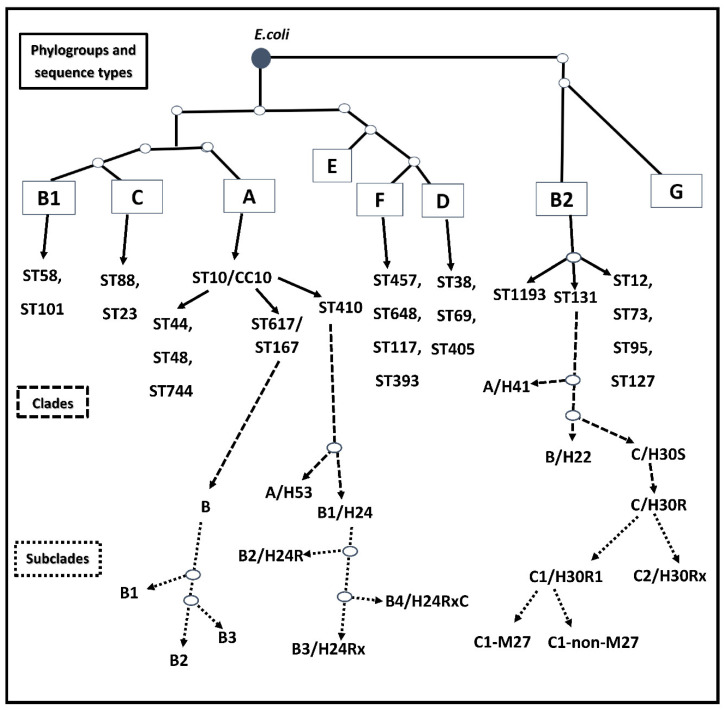
Overview of the phylogenetic groups, sequence types, clades, and subclades of extraintestinal pathogenic *E. coli*.

*E. coli* is classified into eight phylogroups using a PCR-based method, multilocus sequence tying (MLST, Achtman schema), and complete genome data. These phylogroups are A, B1, B2, C, D, E, F and G. This phylogenetic classification of *E. coli* has been applied to compare serogroup, virulence and resistance traits, as well as the distribution of *E. coli* strains among various hosts [33,34,35,36] (Figure 1).

According to genetic markers of *E. coli*, numerous sequence types (ST) and clonal complexes (CC) have been described. Currently, the most frequently reported lineages are ST131, ST69, ST10, ST405, ST38, ST95, ST648, ST73 and ST1193, which have been detected in both hospital- and community-associated infections [37,38,39,40]. A high-risk clone is defined as being globally distributed, associated with multiple antimicrobial resistance determinants, able to colonise and persist in hosts for more than 6 months, capable of effective transmission between hosts, has enhanced pathogenicity and fitness, and is able to cause severe and/or recurrent infections [8]. The remarkable genomic plasticity of *E. coli* enables it to acquire genes of toxins and different virulence determinants that lead to the development of intestinal pathogen *E. coli* (IPEC) strains. Notably, enetoropathogenic *E. coli* (EPEC), enterotoxigenic *E. coli* (ETEC), enteroinvasive *E. coli* (EIEC), enterohemorhagic *E. coli* [41].

In this review, we summarise all available data about multiresistant extraintestinal *E. coli* internationally disseminated high-risk clones. We analyse these clones based on their WGS data and according to reported outbreaks, and we summarise their resistance profiles and genetic markers. The well-known clones already disseminated, as well as recently emerging clones, will be described, and the ‘One Health’ approach will be also discussed. The selection criteria of reference articles in this review were scientific articles that describe WGS data and outbreaks of high-risk multiresistant *E. coli* clones.

## 2. CC/ST131, the Worldwide Predominant High-Risk Clone 

*E. coli* ST131 clone has been disseminated worldwide and is one of the major nosocomial pathogens in hospitals all around the world. This clone also plays an important role of spreading antibiotic resistance [42]. ST131 is characterised by a stepwise diversification, with two main serotypes (O16:H5 and O25:H4), three main clades (A, B and C), and three fimH alleles (41, 22 and 30, respectively), all correlated. Using an alternative taxonomy, ST131 is also classified as subclone H30R or H30Rx according to antibiotic resistance patterns [42,43,44].

Originally, ST131 clone was reported as an O25b:H4 serotype, CTX-M-positive, and ESBL production was commonly detected in this clone from Canada, Korea, India, Kuwait, Lebanon, France, Switzerland, Portugal and Spain. The vast majority of strains in this clone are resistant to several antibiotic groups, namely beta-lactams, aminoglycosides, tetracycline, fluoroquinolones, sulfonamides, chloramphenicol and nitrofurantoin [43,44]. Moreover, in the last 15 years, this clone became the predominant high-risk international clone among ExPEC clinical isolates, as 133 of 169 collected studies have reported its presence from various clinical samples between 1995 and 2018 [15,37,39,45,46,47]. 

The phylogenetic analysis of whole-genome data classified the ST131 clone as the main member of phylogroup B2, which is known as the initial source of diverse sequence types, including ST1680, ST1982, ST1461, and ST1193 [46,48,49,50]. Compared to other *E. coli* phylogroups, B2 is also characterised by its high number of virulence factor-encoding genes [51]. On the other hand, unlike other B2 ExPEC strains, ST131 is still frequently detected as an ESBL producer, and in most cases it is also fluoroquinolone resistant [52].

According to resistance traits and population genetics, ST131’s phylogeny has been clustered into three major clades with well-defined resistance profiles, namely clade A, B and C [15] (Figure 1). In general, clade A/H41 and clade B/H22 are described as smaller subgroups [45,52,53]. However, clade A ST131 strains have been found in many community-associated infections, and these have also been reported in stool samples of healthy children from randomly selected primary schools in a study from Changsha, China [46,54]. Furthermore, clade A *E. coli* strain has been reported in water from the Jurong river reservoir in Singapore. It carried aminoglycoside-transferases and *bla*_CTX-M-27_ with mutations in *gyrA*, *parC* and *parE* [55]. On the other hand, clade B has been described as a foodborne pathogen with the ability to colonise poultry, contaminate meat and express colistin resistance genes (*mcr-1* and *mcr-3*). In the case of human infections, clade B strains have been isolated from urine, blood and peritoneal fluid samples [38,46]. It has been hypothesised that another possible source of strains from clade B is the consumption of vegetables from contaminated soil, as this clade has also been detected in agricultural soil. These strains carried a large resistome, including *mcr-1.1*, *bla*_CTX–M–15_ and *qnrB19*. GenBank Accession number: *JAENHI000000000.1* [56]. (Table 1).

Recently, the most significant clinical problems have been related to clade C. It originates from clade B, and consists of two major subclades, namely C1/H30-R and C2/H30-Rx (Figure 1). Their evolution has been demonstrated, as they arose from an early common fluoroquinolone-susceptible ancestor C/H30 subclone with type 1 fimbrial adhesin gene (*fimH30)*. Initially, H30 was the most prevalent among them, emerging in the 1980s. In the course of clonal expansion, it obtained high-level fluoroquinolone resistance by sequential chromosomal mutations of *gyrA* and *parC* genes, then it also became resistant against beta-lactams by acquisition of plasmid-mediated ESBLs, as well as carbapenemases [15,29,45,52,53,58]. The self-transmissible plasmids of ST131 are characterised by a remarkable genetic diversity (plasmidome), they belong in particular to incompatibility group F (IncF type). They may possess FIA or FII replicon types, which aid in the successful uptake and rapid dissemination of resistance genes. The most frequently reported plasmid MLST types are F1:A2:B20 in multidrug-resistant (MDR) clade C1 and F2:A1:B of MDR clade C2 [15,78,79]. A recent in-depth analysis showed that by a novel subset of C2, the plasmidome was not uniform; it had a combined pattern of certain plasmid types, and it showed a homogeneous replicon structure of F31/F36:A4:B1 [58]. 

In general, a common feature of strains in clade C is the carriage of *bla*_TEM_. However, subclade C1 presents *bla*_CTX-M-14_ or *bla*_CTX-M-27_ ESBL genes, while on the other hand, subclade C2, which has a single nucleotide polymorphism (SNP) at fimH30, is mainly associated with *bla*_CTX-M-15_. The *bla*_CTX-M-27_ positive subset of C1, referred to as subclade C1-M27, recently became prominent in Japan, Canada, Germany and China [20,46,80,81,82]. In Iran, a comparative study on MDR ST131 and non-ST-131 clones reported that both were associated with *bla*_TEM_, *bla*_SHV_, *bla*_CTX-M_, *bla*_OXA-48_ genes, as well as with plasmid-mediated quinolone resistance (PMQR) determinants (bifunctional aminoglycoside acetyltransferase-Ib-cr [*aac6′-Ib-cr*] and Qnr protective proteins [*qnrB*, *qnrS*] [83]. A genomic epidemiological investigation of ESBL producer *E. coli* isolates was also performed in Dhaka, Bangladesh. Not surprisingly, the predominant clone from clinical urine and pus samples was ST131, as this clone accounted for 46% of the isolates. The whole-genome sequences (WGS) of these strains were deposited in GenBank under accession numbers from *JACHQR000000000.1* to *JACHPB000000000.1* [39]. In Brazil a CTX-M-27-producing *E. coli* ST131 strain that belonged to clade C1-M27 was reported in oysters. This *E. coli* strain was recovered from an aquatic area impacted by intensive maritime traffic and transoceanic shipping activities [66]. WGS information is shown in Table 1.

A recent study in Hungary investigated ESBL-producing *E. coli* isolates obtained from a tertiary care hospital in Budapest. Whole-genome sequence analysis showed that five *E. coli* isolates belonged to the ST131 clone: two to the C1-M27 subclade with *bla*_CTX-M-27_ and three to the C2/H30Rx subclade with *bla*_CTX-M-15_. Based on core genome MLST, all C2/H30Rx isolates formed a cluster (≤6 allele differences), while the *bla*_CTX-M-27_-producing C1-M27 isolates differed from each other with respect to at least 35 alleles. This study indicates that the C2/H30Rx and C1-M27 subclades of the ST131 are currently circulating among Hungarian clinical isolates [84].

Carbapenem resistance among ST131 strains is based on plasmid-acquired carbapenemase enzymatic activity [82]. According to a recent genomic epidemiological study that investigated clinical isolates from 62 countries between 2015 and 2017, many subtypes of carbapenemases were carried by ST131. During these studies, ST131 was mainly isolated from UTI and bacteraemia. From two different isolates, *bla*_KPC-3-_ and *bla*_OXA-48_-producer ST131-A clade has been reported in USA and Lebanon, respectively. A subclade C1-M27 was also present, which showed positivity for *bla*_NDM-1_ from Russia and Philippines, and *bla*_OXA-232_ from Thailand. From the C1 subclade, a non-M27 subtype was detected as well, and these strains carried *bla*_KPC-2_ in Guatemala, Israel and USA, *bla*_KPC-3_ in Italy and *bla*_KPC-18_ in the USA. The globally predominant ST131-C2 subclade carried *bla*_KPC-2_ in Puerto Rico, *bla*_KPC-3_ in Italy, *bla*_NDM-1_ in Egypt and *bla*_NDM-5_ in Canada. Ambler class D carbapenemase, namely *bla*_OXA-48_ and *bla*_OXA-181_, production was also described in Egypt and Iran. Additionally, a ST131-C2 strain showed positivity for co-expression of *bla*_NDM-1_ and *bla*_VIM-1._ [40,82]. Located on plasmids with sequence similarities (95–100%), different carbapenemases of ST131 have been detected in other international clones as well [84,85,86]. Additionally, the co-carriage of *bla*_OXA-1_, *bla*_CTX-M-15_, *aac6-Ib-cr* and *aac3-IIa* has also been detected in strains of C2 clade [58,87].

Another recent survey found CC131 subclones in 10 hospitals in different cities in Argentina. The observed samples were mainly blood, urine and abdominal fluids. The majority (7 of 10 samples) belonged to the C2 subclade, and they carried *bla*_KPC-2_, and one strain showed positivity for *bla*_VIM-1_. The C1 subclade was also found, and expressed *bla*_CTX-M−2_. Furthermore, the so called ECO112 KPC-2-producer strain of clade B was fluoroquinolone-susceptible and carried *bla*_FOX−5_. The other isolates of CC131 were resistant to fluoroquinolones based on chromosomal mutations of *gyrA*, *parC* or *parE*, and, additionally, PMQR determinants, namely, *qnrB* and *qnrS1*, were also detected. As an interesting result, a strain referenced as ECO14 of ST131 exhibited resistance to colistin (MIC ≥ 4 µg/mL), but it lacked *mcr.* It developed colistin resistance due to seven chromosomal mutations in the *pmrB* and *pmrA* genes (phosphoetanolamin transferase coding genes) [20].

Moreover, in certain cases, O16:H5 ST131 and rare, even non-typeable relatives were also found. These subclones were compared to the most successful O25b:H4 serotype, and studies detected that the so-called O16 subclone has a higher rate of trimethoprim-sulfamethoxazole and gentamicin resistance, but a lower prevalence of fluoroquinolone and ceftriaxone resistance, than O25b [88]. In addition, a study in Kyoto, Japan described a separated O75:H30 cluster within the C1 subclade, which was characterised by an extraordinary Phi-like region (M27PP1). Subsequently, this ExPEC subtype was reported not only in Japan, but also in Thailand, Australia, Canada and in the USA, so its prevalence has been significantly increased [15,80,89]. 

Finally, the pangenome of clade C is divided into a strongly determined core genome and an additional genetic context with remarkable variability that is responsible for a huge repertoire of virulence factors [83,90]. According to the so-called “perfect storm” theory, acquisition of virulence factors plays an essential role in the clonal expansion of multiresistant bacteria, as the acquirement of these genes is followed by higher antibiotic resistance rates [53]. Based on the PCR-verified presence of certain virulence genes including Afa and Dr adhesins (*afa/draBC*), operon (*afa*), catecholate siderophore receptor (*iroN*), secreted autotransporter toxin (*sat*), *ibeA* (‘invasion of brain endothelium’ gene), allele II and III of *papG* gene (*papGII* and *papGIII*), cytotoxic necrotising factor type 1 (*cnf1*), alpha-hemolysin (*hlyA*), cytolethal distending toxin (*cdtB*) and K1 variant of group II capsule (*neuC-K1*) virotypes are defined from A to E groups [52]. The current evolution of new virotypes in C2 subclade can also be seen, as a study from Singapore demonstrated a monophyletic subclone from bacteraemia referred to as SEA-C2 [58,91].

Interestingly, a comparative genomic analysis of 99 ST131 strains and 40 genomes of other high-risk clones (ST38, ST405 and ST648) showed that clades A, B, and C of ST131 were more distant relatives than the others. This study could not identify any CC131-specific proteins, although the absence of 142 proteins in the core genome of all of the 99 isolates was found. These results suggest that the drive of adaptive strategies of ST131 were mainly loss, exchange, and co-evolution of genes, including that of antimicrobial resistance and virulence [38]. WGS data are available in GenBank (Table 1).

Based on the ‘One Health’ approach, zoonotic risk, as a novel aspect of MDR CC131 global distribution, has been also suggested. As a common feature, many rapid outbreaks of high-risk international MDR bacteria have originated from the human–animal interface [47]. Due to the similar and overlapping molecular regions between avian pathogenic *E. coli* and ExPEC, it has previously been hypothesised that avian *E. coli* may act like a reservoir of virulence and resistance markers. Therefore, it may be responsible for foodborne infections in humans [83,92]. In a study from Iran, ST131 strains from human isolates were compared to isolates obtained from broiler chickens. Half of the isolates from chicken meat belonged to phylogroup A, which exhibited a ciprofloxacin-resistant phenotype, but no ST131 was detected in broilers in that study [83]. On the other hand, studies from Spain, Canada and Arizona confirmed the presence of CC131, mainly clade B, in poultry. Moreover, *mcr-5-* and *mcr-9*-positive strains were also isolated among these ST131 strains [18,57,93,94]. 

A genomic surveillance and cell culture-based virulence investigation study demonstrated the co-presence of *bla*_CTX-M-15_-positive *Klebsiella pneumoniae* ST307 and *bla*_CTX-M-27_-positive CC131 with other phylogroups of ExPEC MDR clones containing various CTX-M types and AmpC in oysters and mussel specimens from the Atlantic Coast of South America. Marine bivalves are filter-feeding organisms, so they can extract a large amount of material from water, such as human faecal pollution, including MDR bacterial strains. Furthermore, production of thermostable toxin has also been reported among these strains, so a great deal of attention should be paid to seafood as a source of diseases induced by high-risk toxin producer MDR bacterial clones. The whole-genome sequence of this ST131 strain was deposited in GenBank under the accession number *NCVZ00000000.1* [47] (Table 1). Furthermore, houseflies have been hypothesised to be vectors of many MDR bacteria, including *Pseudomonas aeruginosa*, *Acinetobacter baumannii*, *Citrobacter freundii*, *Enterobacter cloacae*, *Klebsiella oxytoca* and ExPEC clones, such as CC131, in a tertiary hospital in Rwanda, Africa. This clone carried, among others, *bla*_CTX-M-15_, *bla*_OXA-1_, *bla*_TEM-1B_ and *aac(6′)-Ib-cr.* In this case, randomly captured flies were observed in fly traps over 4 weeks from different locations of the hospital, for instance, from the surgery operating theatre, gynaecology, paediatrics, the restaurant, the kitchen, and the laboratory. Interestingly, ST131 was identified only from the kitchen, and the vast majority of the other MDR species had a similar resistome. The results demonstrated that almost all of them carried *bla*_CTX-M-15_, *bla*_OXA-1_ and some expressed *aac(6′)-Ib-cr* and *qnrB1* [95].

A study from Rwanda investigated 120 ESBL-producing *E. coli* strains from hospitalised patients. Altogether, 30 different sequence types were detected, including pandemic clonal lineage ST131. Frequently found resistance genes included *bla*_CTX–M–15_, *tet*(34), and *aph(6)-Id.* Additionally, a phylogenetic relationship was found among strains from patients and their related community members and animals, indicating transmission of clinically relevant, pathogenic ESBL-producing *E. coli* among patients, animals, caregivers and the community in Rwanda [96].

In summary, the structure of the *E. coli* population has changed dramatically, with appearance and global dissemination of the currently dominant multidrug-resistant C2 subclade of ST131. Nevertheless, from the most successful phylogroup B2, other high-risk clones can evolve and cause alarming challenges too [37].

## 3. ST1193, a Recently Emerging Pandemic MDR Clone from Phylogroup B2

Although ST1193, a sister clone of ST131, had already been described in Australia in 2012, case reports of this clone have increased considerably in number only in the last few years. The ST1193 clone is also known as the latest pandemic multidrug-resistant clonal group [46,97,98,99]. A recent study from France described five *E. coli* strains of ST1193 that were harbouring *bla*_CTX-M-15_ and *bla*_CTX-M-27_. These strains were obtained from febrile urinary tract infections in children [100]. A study from China reported that *E. coli* strains of ST1193 were responsible for more than 20% of neonatal invasive infections in China [100,101]. Furthermore, ST1193 was also found together with clade A ST131 strains in stool samples of healthy children in Changsha, China [46].

One strain of ST1193 was isolated in Dhaka, Bangladesh from a urine sample. It expressed a plasmid-acquired *bla*_CTX-M-15_, and it belonged to the O75:H5 serogroup. The whole-genome sequence data of this strain are available in GenBank under accession number *JACHQB000000000.1* [39]. (Table 1). 

Altogether, 355 strains of ST1193 were investigated in a study in the USA, and various resistance determinants were detected, namely, *bla*_TEM-1B_, *bla*_CMY-2_, *bla*_CTX-M-15_, *bla*_CTX-M-27_, *bla*_CTX-M-55_, *bla*_OXA-1_, *aac(6)′-Ib-cr*, and mutations were detected in genes *gyrA*, *parC* and *parE.* Strains of ST1193 were all lactose non-fermenting and carried *fimH64*, in particular. Its evolutionary development from K1 to K5 capsular types resulted in genomic changes and uptake of an F-type virulence plasmid were also reported [97]. A study from Hungary recently reported a single *E. coli* from clinical isolates that belonged to ST1193 and carried *bla*_CTX-M-27_ [84].

Carbapenem resistance has occurred in ST1193, and *bla*_KPC-2_ and *bla*_NDM-1_ have been reported [40]. Moreover, mutations in *pmrA* and *pmrB* that confer colistin resistance were also confirmed [102]. The complete ST1193 genome from a neonatal meningitis-associated strain is available in GenBank at accession number: *CP030111* [59,97] (Table 1).

## 4. ST69 and CC10, the Second and Third Most Common High-Risk Clones

Overall, based on a comparative summary of 169 studies about ExPEC high-risk clones after the predominant ST131, we found ST69 and ST10 to be the second and third most frequent clones, respectively [37]. Initially, ST69 was isolated in the year 2000, from urine samples of 228 women with uncomplicated community-acquired UTIs at a public university campus of California. ST69 belongs to phylogenetic group D, and it is characterised by diverse O-antigen-based serogroups and the common presence of *papGII*. Since then, most of the reported strains of this clone have been multidrug-resistant, and they typically possess a class I integron that includes a single gene cassette including dihydrofolate reductase and aminoglycoside adenyltransferase (*dfrA17*–*aadA5*). In these samples, a trimethoprim–sulphamethoxazole-resistant Clonal Group A (CgA) was also detected, a clone that clusters within ST69 [103]. Interestingly, based on findings of the phylogenetic features of *E. coli*, it was revealed in England that the *E. coli* population remained stable over time, but some lineages emerged and were disseminated, including ST69 [104]. In total, 87 of 169 studies describe this clone for the period 1995–2018. ST69 is characterised by the presence of *bla*_KPC-2_, *bla*_NDM-1_ [40], co-carriage of *bla*_NDM-1_ with *bla*_CMY-6_, [20], *bla*_CTX-M-1,-14,-15,-27_ [100], *mcr-1* [105], *fosA3* [106] and *gyrA*, *parC* mutations, leading to fluoroquinolone resistance [107]. During other studies in Italy, this clone has also been identified from various origins, including dairy products, the diaphragms of wild boars, poultry, mussels, clams, and human stool. Aside from human specimens, chicken breast carries a wide spectrum of antimicrobial resistance genes [60,108]. The increasing number of cases of this MDR clone indicates the importance of studies of phylogenetics, population dynamics and molecular epidemiology using the ‘One Health’ approach [37].

CC10 belongs to phylogenetic group A, and it has been detected to be a widely disseminated clone, since it has been reported from food producing animals, free-living birds, plant-based foods, retail meats, wastewater, rivers, urban streams, and clinical settings, as well as being a part of human gut microbiome. Thus, faecal carriage in humans probably played a significant role in its clonal expansion and dissemination. This clonal complex is composed of ST10 and its further relatives, including, among others ST44, ST48, ST167, ST617, ST410, and ST744 [37,109,110] (Figure 1). During a survey aiming to characterise the molecular epidemiology of carbapenemase-producing ExPEC in Argentina, CC10 was the major one, accounting for more than 20% of the samples. Of them, eight contained ST10, and the others were single-locus variants (ST44, ST744, ST167), double-locus variants (ST746, ST617) and a satellite clone (ST12667). On the other hand, CC10 was the main clone reported among carbapenemase producers, as it demonstrated positivity (in order of decreasing abundance) for *bla*_KPC-2_, *bla*_NDM-1_ and *bla*_IMP-8_. As an important finding, two of them showed co-expression of *mcr-1*; furthermore, another NDM-1-producer isolate was a co-producer of *bla*_PER-2_. On the other hand, the ST617 clone exhibited the co-existence of *bla*_KPC-2_, *bla*_CTX-M-14_ and *bla*_CTX-M-27_. The nucleotide sequence information was submitted in GenBank under the BioProject accession number *PRJNA784589* [20] (Table 1). In addition to β-lactam resistance, CC10 has also been marked by fluoroquinolone-resistance determinants (e.g., *qnrS1*, *aac(6′)-Ib-cr)* and the *mcr-1 colistin-resistance gene* [7,60,111,112]. In addition, *bla*_OXA-48_ associated with ST10; *bla*_NDM-1_ related to ST44, ST48, ST167, ST617; *bla*_CTX-M-14,15,55_*, fosA3, bla*_OXA-1_, *bla*_NDM-1,9_, *bla*_NDM-5_ together with *bla*_OXA-181_ and co-carriage of *bla*_OXA-48_ in ST167; as well as *bla*_KPC-2,3_ with *bla*_NDM-1_ in ST617 have also been reported. Of these high-risk CC10 lineages, in the last few years, ST167 was clustered into subclades, and it was reported to be a predominant clone in China. This clone was also identified from a urine clinical sample as a *qepA4* carrier [7,40,61,62,112,113].

Several studies have been reported indicating the high risk potential of ST410 from phylogenetic group A [5] (Figure 1). This clone has been described in many countries, albeit to a lesser extent compared to other high-risk clones. ST410 has been reported to be a clone that is transmitted between different reservoirs, namely, between wildlife, humans, companion animals, and the environment [114,115]. The ST410 clone has been reported as being *bla*_OXA-181_ positive in China [116] and Italy [117], as well as hospital outbreaks in Denmark [118]. A study from Dhaka, Bangladesh reported ST410 as being *bla*_CTX-M-15_ positive [39]. 

A whole-genome sequence analysis of *E. coli* ST410 in Denmark revealed carriage of *bla*_OXA-181_ and *bla*_NDM-5_ on IncX3 and IncF plasmids, respectively [119].

## 5. ST405 and High-Risk CC/ST38 Clones from Phylogenetic Group D

ST405 is a globally reported clone that carries similar variants of virulence genes to O25b:H4 ST131 [65]. Recently, this clone was marked as a potential reservoir for *bla*_NDM-5_ [40,62,119]. NDM-5-producing ST405 has been detected in many geographic regions, but it has shown the highest prevalence in the United Kingdom and Italy [63,120,121]. 

Moreover, an autochthonous case in 2018 was reported in Italy. The isolated strain carried *bla*_NDM-5_, and among others *bla*_CMY-42_, *aadA2*, *mdf(A)*, *sul1* and alterations of *gyrA, parC*, *parE* [121]. The presence of *bla*_NDM-5_ has also been detected in Japan and Mozambique, Africa [63,64]. This clone in Japan was non-susceptible to fluoroquinolones and β-lactams, including broad-spectrum cephalosporins and carbapenems, but it kept its susceptibility against tigecycline. The complete genome sequence of this strain is available under BioProject number: *PRJDB8512* [63] (Table 1).

The O102:H6 serotype was reported in Mozambique for the first time, possessing an FI:A1:B49 plasmid that co-harboured *bla*_NDM-5_, *bla*_CTX-M-15_, *bla*_TEM-1_, *aadA2*, *sul1* and *dfrA12* genes. Additionally, this strain had chromosomal mutations of *gyrA, parC* and *parE*, resulting in fluoroquinolone resistance. The WGS data of this strain are available in the EMBL-EBI database, project accession number: *ERS4552076* [64] (Table 1).

Another Ambler class B beta-lactamase, namely *bla*_NDM-4_, was also identified on a mosaik IncFIA-type plasmid in three ST405 strains (GenBank accession numbers: *NGUU00000000*, *NGUV00000000* and *NGUW00000000*) [65] (Table 1). These strains carried diverse resistance determinants, *bla*_CTX-M-15_, *bla*_OXA-1_, *aac(6′)-Ib-cr*, *aac(3)-IIa*, *aadA5*, *strA*, strB, *tet(A)*, tet(B), sul1, *sul2*, and dfrA17. However, all of these ST405 strains were susceptible to colistin [64]. Further carbapenemases, such as OXA-48 and KPC-2, have also been reported in ST405 [40].

A study from Algeria reported *mcr-1* in ST405 *E. coli* in environmental samples taken from eight agricultural sites in North West Algeria [122].

Interestingly, a study identified a novel mobile *IS*26-flanked transposon in the chromosome of the high-risk ST405 clone. Resistance genes were carried by a chromosomally integrated class 1 integron with *dfrA17* and *aadA5* gene cassettes. Its nucleotide seqeunce was deposited in GenBank under the accession number *NXER00000000* [65] (Table 1). A study from Japan reported *bla*_TEM−1A_, *bla*_OXA−1_, *bla*_CTX−M−14,15,24_, *aac(6′)-Ib-cr* in *E. coli* ST405 [123]. 

Similar to ST405, CC/ST38 was also previously a neglected clone, but nowadays it belongs to the so-called ‘significant minority’ of ESBL-producer *E. coli*, accounting for approximately 12% of strains from UTI [37,38,69]. Compared to ST131, the phylogenetic background of ST38 is far less detailed. It has mainly been described, on the basis of various O:H serotypes, as a hybrid uropathogenic–enteroaggregative clone [68,124].

During one study, three multiresistant *E. coli* strains were detected from rectal samples taken in the course of screening from three patients in Paris, France. Two patients had stayed previously in Egypt, and the third patient had come from Turkey. All three *E. coli* isolates belonged to the ST38 clone, and showed resistance to penicillins, cefotaxime, sulfonamides, tobramycin, and gentamicin, but remained susceptible to amikacin, tetracycline and fluoroquinolones. They also demonstrated a reduced susceptibility to carbapenems based on the presence of *bla*_OXA-48_-harbouring plasmid. Co-carriage of *bla*_CTX-M-2_, a point mutant variant of CTX-M-14, and *bla*_TEM-1_ was reported in that strain. Furthermore, this study demonstrated that these strains were clonally related; the same clonal strain probably circulated in Turkey and Egypt, and was later introduced into France [125]. 

Further studies have reported *bla*_CTX-M-3,-9,-14,-15_, *bla*_CMY-12_, *bla*_TEM-1B_, *bla*_NDM-1,6_ and *bla*_OXA-48_ in *E. coli* ST38 [40,68,69,100,126,127,128]. Several outbreaks caused by *E. coli* ST38 carrying a *bla*_OXA-244_ (a point-mutation derivative of *bla*_OXA-48_) in Germany have also been reported [129].

On the other hand, *bla*_CTX-M-14_ and bla_CTX-M-27_ genes were also found in ST38, including in surveys from Germany, Switzerland and the USA [68,69,128]. Moreover, the *bla*_CTX-M-27_ gene was encoded by two distinct plasmid variants. In ST38, it was encoded in an IncF(F2:A-:B10) plasmid; by contrast, in ST131, it was located on IncF(F1:A2:B20) [68,69]. During this study in New York, USA the ST38 strains co-carried *bla*_OXA-48_, *bla*_DHA-1_ and *bla*_CTX-M-14_. The sequence information is available under BioProject accession numbers *PRJNA692174* and *PRJNA510429* [68] (Table 1). Moreover, MDR ST38 strains are often characterised as having a higher number of alterations in nitroreductase genes (*nfsA* and *nfsB)*, resulting in nitrofurantoin resistance. The accession number for GenBank is *RZEE00000000* [69] (Table 1). ST38 has also been detected as a colistin-resistant clone carrying *mcr-5* in healthy chickens in a farm in Paraguay [130].

A study from Japan found that the *bla*_NDM-1_ gene was embedded between two *IS903* elements as a gene cassette in an IncA/C-type plasmid. This transposon region was compared to plant pathogen bacteria, and homologous sequences were identified indicating that these microbes (e.g., *Pseudoxanthomonas* and *Xanthomonas* spp.) are potential sources of the *bla*_NDM-1_ gene [131]. In addition to the relationship between plant pathogens and ST38, this clone was also identified in Mongolian birds, but the acquired ESBLs (*bla*_CTX-M-14_ and *bla*_CTX-M-15_), independently of the antimicrobial selective pressure, were stably harboured by their chromosome instead of plasmids [132]. In addition, CC38 and CC10 were the predominant pandemic STs in food and among environmental *E. coli* strains in Brazil during a recent genomic surveillance analysis [112].

## 6. ST457, a Novel Emerging Clone from Phylogroup F

ST457 was first described in 2008 in the United Kingdom, and it was obtained in a clinical isolate from UTI. However, since then, the *E. coli* ST457 clone has emerged as a diverse *E. coli* clone present on all continents and from various samples, even in wild animals from Antarctica [19,133]. As evidence for further possible zoonotic and zooanthroponotic (reverse zoonotic) linkages, close similarities were found between Australian human clinical and silver gull strains among the H45 clade of ST457 [19]. These strains are characterised by carbapenemase production in patients with sepsis, namely *bla*_KPC-2_ (from Italy and Mexico), *bla*_KPC-3_ (from USA), *bla*_NDM-5_ (from Shanghai), and *bla*_IMP-4_ were also described in Australia, and interestingly, *bla*_NDM-9_ was detected in a poultry specimen. Surprisingly, chromosomally located *bla*_OXA-23_ was identified from Australian gull samples too [19,134,135,136,137]. Additionally, this lineage often carries further beta-lactam resistance genes, the most common of which is *bla*_CMY-2_ from AmpC β-lactamases. ESBLs were also detected in ST457, including *bla*_CTX-M-1,-2,-3,-8,-12,-14,-15,-27,-55_ [19]. 

Colistin resistance has been reported in the ST457 clone in many cases. This can be explained by the presence of plasmid-mediated *mcr-1* from human clinical isolates in the USA, China, Vietnam, Mexico, and from wildlife and poultry in Asia [138,139,140,141]. Furthermore, an *mcr-3* variant was also identified in ST457 from a single domestic duck in China [142]. In addition, located on transferable plasmids, *mcr-5*, *bla*_CTX-M-8_, *bla*_TEM-1A_, co-expression of *aph*(*6*)-*Id aph-Ib*, and *sul2* were found among healthy chickens [19,70,130]. GenBank Accession numbers for high-risk international ST457 clones are available in Table 1.

## 7. Further Potential ExPEC High-Risk Clones

Several additional *E. coli* clones from various phylogenetic groups are also clinically relevant ExPEC clones, as they are responsible for an enormous burden of extraintestinal infections, including sepsis, UTI and neonatal infections across the globe. These addition-al clones are ST95, ST73, ST12, ST127 (from phylogroup B2), ST117, ST393, ST648 (from phylogroup F), and CC23/ST88 (from phylogroup C) [20,37,38,39,40,46,71,72,74,75,83,100,103,104,112,133,143,144] (Figure 1).

The current dissemination of ST58 and ST101 from phylogenetic group B1 is alarming, because previously, this group was reported to be a cluster of environmental bacteria. However, recently, these clones have been described as causative agents of invasive infections (e.g., bloodstream infections). Interestingly, these clones have not been reported to be carbapenemase producers or as being colistin resistant, yet [76,77]. Currently, there are only a small number of reports available about these clones that explain their emergence and dissemination [20,37,38,39,40,46,83,112,130,133,143,144,145]. The related genome-sequence information from published reports is summarised in Table 1.

## 8. Discussion

The emergence, expansion, and recent outbreaks of ExPEC high-risk international clones are of great concern worldwide [37,40,106]. MDR high-risk ExPEC is commonly detected in both nosocomial and community-acquired infections, and these infections are usually difficult to treat, because therapeutic options are limited [37,38,39]. The genome of *E. coli* has plasticity and high variability, and therefore various resistance and virulence genes can be taken up from different species of *Enterobacterales* and can be passed on to other species [19,95]. The development of these MDR strains depends significantly on the features of a given geographic area, such as trends of antibiotic consumption, resistance profile among currently circulating pathogens, travelling habits, medical tourism, and previous hospitalisation [64,121,125]. Furthermore, in countries such as Canada, the USA, Korea, Kuwait, Lebanon, France, Switzerland, Portugal, Spain, Germany, Bangladesh, China, and Japan, many lineages (e.g., ST131) circulate with quite similar resistance patterns [8] (Table 2).

Globally, the predominant ExPEC clone is ST131, which is clustered into clades and subclades [15,37,39,45,46,47]. It possesses a wide variety of beta-lactamases, with the most frequent including ESBLs (*bla*_CTX-M-14,15,27_), AmpCs (*bla*_FOX−5_, *bla*_DHA-1_), MBLs, and carbapenemases (*bla*_NDM-1,5;_ *bla*_VIM-1_, *bla*_KPC-2,-3,-18;_ *bla*_OXA-48,-181,-232_). Carbapenem resistance through acquisition of *bla*_NDM-1_ or other variants, namely *bla*_NDM-4,5,6,9_, has been reported in all clones described in this review. Interestingly, *bla*_OXA-48_ and its variants (e.g., OXA-244) have also been disseminated with an increasing tendency in the last few years, as they were detected in CC10, ST405 and ST38 [40,129]. By contrast, *bla*_IMP-8_ was identified only among CC10 strains [20]. Additionally, a point mutation derivate of *bla*_CTX-M-14_, namely *bla*_CTX-M-2_, also appeared in the ST38 and ST457 strains [19,125]. All clones in this review article have a close to equal number of plasmid-mediated ESBLs and carbapenem-clefting enzymes, but they showed significantly lower capacity for AmpCs (Table 2). 

Furthermore, in most cases, the observed high-risk clones also showed resistance to fluoroquinolones, based on chromosomal mutations in *gyrA*, *parC*, *parE* and/or expression of *qnrA1*, *qnrB4*, *qnrB19*, *qnrB66*, *qnrS1* and *aac(6′)Ib-cr* [55,56,58,83,107,112]. In general, single alterations of GyrA and ParC/ParE resulted in a high level of fluoroquinolone resistance among the investigated clones, while low-level resistance occurred with the carriage of Qnr protective proteins (*qnrB19*, *qnrS1*) or *aac(6′)Ib-cr* enzymatic activity. However, double alterations of *gyrA* (S83L, D87N) and *parC* (S80I, E84V) in ST131, CC10, ST38, ST58 were also found. In addition, *qepA4* efflux pump associated with double *gyrA* mutations and an amino acid-exchange of *parC* was identified in ST167 (CC10), as well. Interestingly, based on the available data, the rarely reported clones (e.g., ST393, ST3024, ST354) had a higher potential for genetic changes in *gyrA* and *parC*. As a typical feature, ST744 possessed double *gyrA* (S83L, D87N) and double *parC* (S80I, A56T) alterations [112].

**Figure 2:**
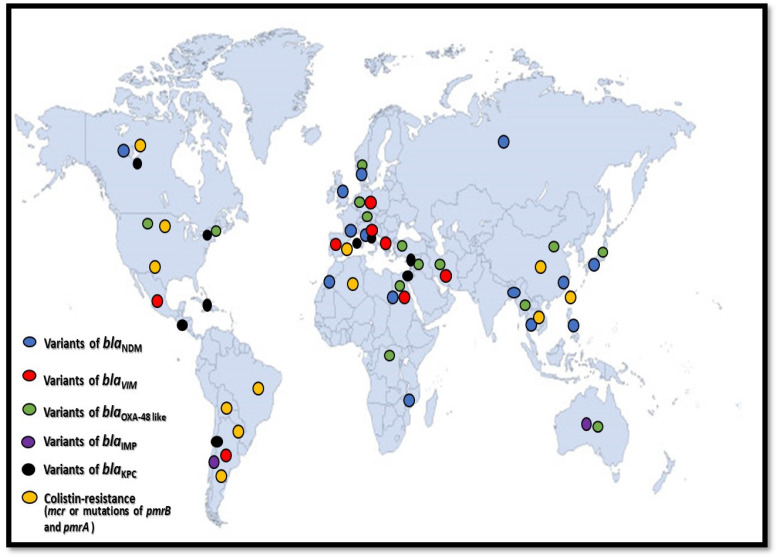
Geographic dissemination of the most frequently reported carbapenemases and colistin resistance among the extraintestinal pathogenic high-risk international *E. coli* clones, http://www.intrafor.com/locations-worldwide-presence.html (accessed on 24 November 2022).

In this comparative analysis we investigated the properties of colistin resistance among these clones. This is an important issue, since polymyxins are considered to be the last resort of antibacterial agents against MDR *E. coli* [146]. Several clones harbour chromosomal *pmrA* and *pmrB* mutations and plasmid-mediated colistin resistance determinants (*mcr-1,-3,-5,-9*) [7,18,19,20,38,56,57,60,102,105,106,111,130,141,142]. In addition, apart from the most frequently detailed ExPEC clones (ST131, ST69, CC10, ST405, ST38, ST457, ST410, ST48, ST58, ST88), a huge number of currently less widely known strains carry *mcr*-genes, such as ST57, ST156, ST224, ST345, ST393, ST850, ST3024, ST8900, ST12657 (Table 3 and Table 4, Figure 2).

Furthermore, the majority of these genes are primarily associated with *E. coli* strains from zoonotic (e.g., poultry) and environmental sources (aquatic reservoir). Based on the ‘One Health’ approach, these strains may play a central role in human MDR infections in the future [106,112].

Fosfomycin is also mentioned as being among the last resort antibiotics that has retained antibacterial efficacy against MDR *E. coli* strains [10]. Of great concern, however, numerous fosfomycin-resistant *E. coli* clones have already been reported across the globe [106,112]. In the most common cases, resistance to fosfomycin is based on the enzymatic activity of *fosA3*. In addition, an ST131 clone was detected among patients of a hospital in China that carried *mcr-3* and *fosA3* together on an IncP plasmid [106]. Nitrofurantoin has also been considered to be an option for therapy in the case of UTI caused by MDR *E. coli*. However, resistance to nitrofurantoin has also been reported in ST131 and ST38 [44,69] (Table 3). Although in this study we focused on beta-lactams, fluoroquinolones and colistin, notably, almost all of the studied high-risk clones harboured a multicoloured collection of aminoglycoside-modifying enzymes, sumetrolim, tetracycline resistance genes [107].

Future actions that can be used to investigate and to analyse high-risk extraintestinal *E. coli* clones include surveillance on a genomic level, and the application of databases to detect new emerging clones and resistance determinants [147,148,149,150,151,152,153,154]. In terms of medical importance, novel antibiotics are needed to treat infections caused by multiresistant *E. coli* [21].

In conclusion, antibiotic resistance poses as an ongoing challenge worldwide and high-risk *E. coli* clones play a central role in the dissemination of resistance determinants. Taking the ‘One Health’ approach, high-risk *E. coli* clones circulate among different sources (human, animal, environmental); therefore, WGS analysis is a very useful approach [147,148] for tracking and understanding the changes in population dynamics, detecting resistance determinants and analysing the emergence of novel high-risk international clones.

## Figures and Tables

**Table 1:** Whole-genome sequences of internationally disseminated *E. coli* sequence types from published reports.

Sequence Type	Accession Numbers (Bioproject, Biosample, SRA, European Nucleotide Archive, EMBL-EBI Database Project)	Reference
**ST131-B**	*JAENHI000000000.1* (*E. coli* strain S802)	[56]
	*VEWQ00000000* (*E. coli* strain UPEC U34)	[57]
**ST131-C**	*JSXN00000000* (*E. coli* strain NA101) *JSXO00000000* (*E. coli* strain NA112)	[38]
	*JACHQR000000000.1* (*E. coli* strain LMLEEc001) *JACHQP000000000.1* (*E. coli* strain LMLEEc003) *JACHQO000000000.1* (*E. coli* strain LMLEEc010) *JACHQK000000000.1* (*E. coli* strain LMLEEc025)	[39]
	*JAJPAL000000000.1* (*E. coli* strain ECO112)	[20]
	*NCVZ00000000.1* (*E. coli* strain EcMO)	[47]
	*PRJEB46895* (*E. coli* strain EC-119)	[58]
**ST1193**	*CP030111* (*E. coli* strain MCJCHV-1)	[59]
	*JACHQB000000000.1* (*E. coli* strain LMLEEc041)	[39]
**ST69**	*SAMN11246379 (E. coli strain EC81)* *SAMN11246556 (E. coli strain EC369)* *SAMN11246590 (E. coli strain EC801)* *SAMN11246609* (*E. coli* strain EC820)	[60]
**CC10 (ST617)**	*PIZJ00000000* (*E. coli* strain ECCO2)	[61]
**CC10 (ST167)**	*CP074120* (*E. coli* strain EC16)	[7]
	*QLNK00000000* (*E. coli* strain ECWJ1)	[62]
**ST405**	*AP019803* (*E. coli* strain KY1497)	[63]
	*ERS4552076 (E. coli strain* SSM100)	[64]
	*NGUU00000000* (*E. coli* strain WCHEC96200)	[65]
	*NXEQ00000000* (*E. coli* strain 2009-30) *NXER00000000* (*E. coli* strain 2009-27)	[66]
**ST38**	*NCWA00000000.1* (*E. coli* strain Ec6M)	[67]
	*SAMN17315482* (strain URMC_401_E_coli)	[68]
	*RZGB00000000* (*E. coli* strain URMC_9) *RZBC00000000* (*E. coli* strain URMC_13)	[69]
	*NCWA00000000.1* (*E. coli* strain Ec6M)	[47]
	*MVIO00000000* (*E. coli* strain NA090)	[38]
**ST457**	*NDBC00000000* (*E. coli* strain EM1CRO)	[70]
**CC23**	*NBCL00000000* (*E. coli* strain 13B) *LYPE00000000* (*E. coli* strain Ec47VL)	[71]
**ST95**	*CP012625* (*E. coli* strain SF-468) *CP012631* (*E. coli* strain SF-173) *CP012633* (*E. coli* strain SF-166) *CP012635* (*E. coli* strain SF-88)	[72]
**ST73**	*JACHPD000000000.1* (*E. coli* strain LMLEEc115)	[39]
**ST410**	*VFBH01000000* (*E. coli* strain A240)	[73]
**ST648**	*PEDQ00000000.1* (*E. coli* strain ICBECG2) *PEDR00000000.1* (*E. coli* strain ICBECG4)	[74]
**ST393**	*QGIF00000000* (*E. coli* strain 77H) *QHCX00000000* (*E. coli* strain 51H)	[75]
**ST58**	*SRX10825685* (*E. coli* strain IBIS_39)	[76]
**ST101**	*VYQD00000000* (*E. coli* strain EC121)	[77]

**Table 2:** Overview of the most common resistance genes against beta-lactams, fluoroquinolones and colistin among high-risk international ExPEC clones. The most frequently reported plasmid types are also summarised here.

Resistance Markers of ExPEC High-Risk Clones						
Sequence Type (ST) [References]	Beta-Lactamases			Resistance to Fluoroquinolones	Resistance to Colistin	Inc Plasmid Types
	ESBL	AmpC	Carbapenemases (Ambler Class A, B, D)			
**ST-131-A** [46,54,55]	*bla*_CTX-M-14_, *bla*_CTX-M-15_, *bla*_CTX-M-27_**+**,	*bla*_DHA-1_	*bla*_KPC-3_, *bla*_OXA-48_	mutations in *gyrA*, *parC*, *parE*, double alteration of *gyrA +*, *qnrB4*, *qnrB66*, *aac(6′)Ib-cr*	**No data available**	FI, FII, A1, B1, B10
**ST-131-B** (*) + [38,46,56,57]	*bla*_CTX-M-15_ **+**	**No data available**		*qnrA1*, * qnrB19**+**	*mcr-1*,(*) *mcr-3*,(*) *mcr-5*, * mcr-9*	F-ColV like lineage, FIB-like, FII, HI2, HI2A
**ST-131-C1-M27** [20,46,83]	*bla*_CTX-M-27_, *bla*_CTX-M−2_, *bla*_TEM_	**No data available**	*bla*_NDM-1_, *bla*_OXA-232_	**No data available**		FI:A2:B20
**ST-131-C1-non-M27** [58]	*bla*_CTX-M-14_, *bla*_TEM_	**No data available**	*bla*_KPC-2_, *bla*_KPC-3_, and *bla*_KPC-18_	**No data available**		
**ST-131-C2** (*) [40,58]	*bla*_CTX-M-15_, (*) *bla*_CTX-M-G-1_, *bla*_CTX-M-G-2_, *bla*_CTX-M-G-8_, *bla*_CTX-M-G-9_, *bla*_CTX-M-G-25_, *bla*_TEM_ (*) *bla*_SHV-12_	*bla*_FOX−5_,	*bla*_NDM-1_, *bla*_NDM-5_, *bla*_VIM-1_, *bla*_KPC-2_, *bla*_KPC-3_, *bla*_KPC-18_, *bla*_OXA-48_, *bla*_OXA-181_, *bla*_OXA-232_, co-expression of *bla*_NDM-1_ and *bla*_VIM-1_	*qnrB19*, (*) *qnrS1*, mutations in *gyrA, parC, parE*	mutations in *pmrA* and *pmrB*, *mcr-3*	FII:A1:B-, F36:A4:B1
**ST1193** [39,40,59,84,102]	*bla*_CTX-M-9_, *bla*_CTX-M-15_, *bla*_CTX-M-27_, *bla*_CTX-M-55_, *bla*_TEM-1B_	*bla*_CMY-2_	*bla*_NDM-1_, *bla*_KPC-2_	mutations in *gyrA, parC* or *parE*, *aac(6′)Ib-cr*	mutations in *pmrA* and *pmrB*	FII, FIB, I1
**ST69** (*) [20,40,60,100,105]	*bla_CTX-M-1_*, *bla_CTX-M-14_*, *bla_CTX-M-15_*, *bla_CTX-M-27_*, *bla*_TEM-1B_, (*) *bla*_TEM-1C_	*bla*_CMY-6_ (co-carriage with *bla*_NDM-1_)	*bla*_NDM-1_, *bla*_KPC-2_	mutations in *gyrA* and *parC* (*)	*mcr-1,+* *mcr-3+*	FIIB
**CC10 (containing ST10, ST44, ST48, ST167 and ST617)** **(+)** [7,61,62,112,113]	*bla*_CTX-M-14_, *bla*_CTX-M-15_, *bla*_CTX-M-27_, *bla*_CTX-M-55_, *bla*_TEM-1B_, *bla*_TEM-169_, *bla*_TEM-206_, *bla*_TEM-214_ *bla*_SHV-12_	*bla*_CMY-42_,	*bla*_NDM-1_, *bla*_NDM-5_, *bla*_NDM-9_, *bla*_IMP-8_, *bla*_KPC-2_, *bla*_KPC-3_, *bla*_OXA-181_, (co-expressed with *bla*_NDM-5_) *bla*_OXA-48_ (co-expressed with *bla_NDM-5_*)	mutations in *gyrA* and *parC*, *qnrS1*, (*) *qnrB19*, *qepA4*, *aac(6′)-Ib-cr*	*mcr-1* (co-expressed with *bla_NDM-1_*)	IncFIA, Col-like, I1
**ST405** **(+)** [63,64,65,66]	*bla*_CTX-M-14_, *bla*_CTX-M-15_, *bla*_CTX-M-88_, *bla*_TEM-1_, *bla*_TEM-12_	**No data available**	*bla*_NDM-4_, *bla*_NDM-5_, *bla*_KPC-2_, *bla*_OXA-48_	mutations in *gyrA, parC*, and *parE*, *qnrS1*, *aac(6′)-Ib-cr*	*mcr-1*+	FIB, FII, F1A, Col-like, F1:A1:B49
**ST38** (*) [38,40,67,68,69,70]	*bla*_CTX-M-2_, *bla*_CTX-M-3_, *bla*_CTX-M -9_, *bla*_CTX-M -14_, (*) *bla*_CTX-M-15_, (*) *bla*_CTX-M-27_, *bla*_TEM-1B_	*bla*_CMY-12_, *bla*_DHA-1_	*bla*_NDM-1_, *bla*_NDM-6_, *bla*_OXA-48_, *bla*_OXA-244_	mutations in *gyrA*, *parC*	*mcr-5* (*)	FII:A-:B10, FI:A2:B20
**ST457** (*) [19,70]	*bla*_CTX-M-1_, *bla*_CTX-M-2_, *bla*_CTX-M-3_, *bla*_CTX-M-8;_ *bla*_CTX-M-12_, *bla*_CTX-M-14_, *bla*_CTX-M-15_, *bla*_CTX-M-27_, *bla*_CTX-M-55_, *bla*_TEM-1_	*bla*_CMY-2_	*bla*_NDM-5_, *bla*_NDM-9_, *bla*_IMP-4_, *bla*_KPC-2_, *bla*_KPC-3_, *bla*_OXA-23_	mutations in *gyrA* and *parC*, *qnrB19* *	*mcr-1* (*), *mcr-3* (*), *mcr-5* (*)	F64:A:B:27, I1

* Clones or genes with zoonotic origin; + Clones or genes with environmental origin; (*) referenced resistance genes isolated from both human and zoonotic origin; (**+**) referenced resistance genes isolated from both human and environmental origin.

**Table 3:** Distribution of colistin, fosfomycin and nitrofurantoin resistance determinants among high-risk international ExPEC clones from published reports. The most frequently reported clones are highlighted in red.

Linkage between ExPEC Clones and Colistin, Fosfomycin, Nitrofurantoin Resistance Determinants						
Colistin Resistance					Fosfomycin Resistance	Nitrofurantion Resistance
*pmrA* and *pmrB* Mutations	*mcr-1*	*mcr-3*	*mcr-5*	*mcr-9*	*fosA3* Enzymatic Activity	Nitroreductase (*nfsA*, *nfsB)* Enzymatic Activity
**ST131** **ST1193**	**ST131** **ST69** **CC10** **ST405** **ST457** **ST410** **ST48** **ST58** **ST88** **ST57** **ST156** **ST224** **ST345** **ST354** **ST393** **ST744** **ST850** **ST3024** **ST8900** **ST12657**	**ST69** **ST457** **ST101** **ST155** **ST206** **ST443** **ST1081** **ST1638** **ST5038**	**ST131-B** **ST38** **ST457** **ST57** **ST93** **ST113** **ST165** **ST189** **ST224** **ST366** **ST580** **ST641** **ST752** **ST2705** **ST6853** **ST8061**	**ST131-B**	**ST131** **ST1193** **ST69** **CC10** **ST38** **ST457** **ST95** **ST12** **ST117** **ST307** **ST648** **ST744** **ST746** **ST1730** **ST2646**	**ST131** **ST38**

**Table 4:** Chronology of evolution of high-risk international *E. coli* clones. The table contains the year of isolation for each clone.

Resistance Genes and Mechanisms										
Clones	*bla_CTX-M-14-_*Variants (e.g., CTX-M-2)	*bla*_CTX-M-15_	*bla*_CTX-M-27_	*bla*_KPC_-Variants	*bla_OXA-48-_*Variants (e.g., OXA-244)	*bla*_IMP-_Variants	*bla*_NDM_-Variants	*bla*_VIM-_Variants	Fluoro-Quinolones	Colistin
**ST131 (including A, B, C1, C2 clades)**	2004	2005	2004	2011	2012		2010	2015-2017 (2022) ^1^	2004	2014
**ST1193**		2013	2017	2022			2022		2012	2021
**ST69**	2014–2017 (2020) ^2^	2014–2017 (2020) ^2^	2014–2017 (2020) ^2^	2015–2017 (2022) ^1^			2022		2017	2018
**CC10 (ST10 with its variants, namely ST167, ST617, ST410)**	2015	2015	2017	2016	2015	2022	2015		2017	2022
**ST405**	2012	2012		2016	2016		2013 (2020), 2016 ^3^		2012	2020
**ST38**	2011	2020	2020		2011		2011		2013 (2015) ^4^	2019
**ST457**	2016	2016	2012	2014		2020	2019	2014	2008	2012 (2020) ^5^

Legend: 1. Peirano et al. summarised carbapenemases including *bla*_VIM_ for the period 2015–2017 [40]. 2. Birgy et al. summarised ESBLs for the period 2014–2017 [100]. 3. The strain was isolated in 2013, and further investigation was performed in 2020; the other strain that contained both of NDM-5 and OXA-48 was described in 2016. 4. The strain was isolated in 2013 (between December 2012 and January 2013), and further investigation was performed in 2015. 5. The strain was isolated in 2012, and further investigation was performed in 2020.

## Data Availability

Not applicable.
